# Conundrum of Classifying Subtypes of Pulmonary Hypertension—Introducing a Novel Approach to Classify “Borderline” Patients in a Population with Severe Aortic Stenosis Undergoing TAVI

**DOI:** 10.3390/jcdd9090294

**Published:** 2022-09-04

**Authors:** Elke Boxhammer, Sarah X. Gharibeh, Bernhard Wernly, Malte Kelm, Marcus Franz, Daniel Kretzschmar, Uta C. Hoppe, Alexander Lauten, Michael Lichtenauer

**Affiliations:** 1 Department of Internal Medicine II, Division of Cardiology, Paracelsus Medical University of Salzburg, Müllner Hauptstraße 48, 5020 Salzburg, Austria; 2 Department of Internal Medicine, General Hospital Oberndorf, Teaching Hospital of the Paracelsus Medical University Salzburg, 5110 Oberndorf, Austria; 3 Division of Cardiology, Pulmonology, and Vascular Medicine, Medical Faculty, University Düsseldorf, 40225 Düsseldorf, Germany; 4 Universitätsherzzentrum Thüringen, Clinic of Internal Medicine I, Departement of Cardiology, Friedrich Schiller University, 07737 Jena, Germany; 5 Department of General and Interventional Cardiology and Rhythmology, Helios Clinic, 99084 Erfurt, Germany; 6 Deutsches Zentrum für Herz-Kreislauf-Forschung (DZHK), Standort Berlin, 10115 Berlin, Germany

**Keywords:** aortic valve stenosis, pre-capillary, post-capillary, pulmonary hypertension, right heart catheter, TAVI

## Abstract

Background: Transcatheter aortic valve implantation (TAVI) is an established therapeutic option in patients with severe aortic valve stenosis (AS) and a high surgical risk profile. Pulmonary hypertension (PH)—often co-existing with severe AS—is associated with a limited factor for prognosis and survival. The purpose of this study was to evaluate the prevalence of PH in patients undergoing TAVI, classify these patients based on right heart catheter (RHC) measurements in different PH subtypes, and analyze prognostic values on survival after TAVI. Methods: 284 patients with severe AS underwent an RHC examination for hemodynamic assessment prior to TAVI and were categorized into subtypes of PH according to the 2015 European Society of Cardiology (ESC) guidelines. TAVI patients were followed-up with for one year with regard to 30-days and 1-year mortality as primary endpoints. Results: 74 of 284 participants showed a diastolic pressure gradient (DPG) < 7 mmHg and a pulmonary vascular resistance (PVR) > 3 Wood units (WU) and could not be formally allocated to either isolated post-capillary PH (ipc-PH) or combined pre- and post-capillary PH (cpc-PH). Therefore, a new subgroup called “borderline post-capillary PH” (borderlinepc-PH) was introduced. Compared with TAVI patients with pre-capillary PH (prec-PH), ipc-PH patients suffering from borderlinepc-PH (HR 7.114; 95% CI 2.015–25.119; *p* = 0.002) or cpc-PH (HR 56.459; 95% CI 7.738–411.924; *p* < 0.001) showed a significantly increased 1-year mortality. Conclusions: Postcapillary PH was expanded to include the so-called “borderlinepc-PH” variant in addition to the ipc-PH and cpc-PH subtypes. The one-year survival after TAVI was significantly different between the subgroups, with the worst prognosis for borderlinepc-PH and cpc-PH.

## 1. Introduction

The prevalence of AS as the leading valvular heart disease in elderly patients is expected to increase due to an aging population over the next decades [1]. TAVI—an alternative minimal-invasive therapeutic option to surgical valve replacement—is well-established in different risk populations [2]. PH is a common comorbidity in patients with symptomatic severe AS and is an established predictor of increased morbidity and mortality in patients receiving TAVI [3].

In the guidelines of the ESC [4], PH is defined by a mean pulmonary arterial pressure (mPAP) of 25 mmHg and occurs in various diseases with different underlying pathomechanisms. To classify PH in subtypes, left ventricular end-diastolic pressure (LVEDP), reflected by the mean pulmonary capillary wedge pressure (mPCWP), plays a major role. Diseases of pulmonary vessels or the lungs themselves primarily cause prec-PH and are defined with a mPCWP ≤ 15 mmHg, whereas diseases of the left heart cause post-capillary PH with a mPCWP > 15 mmHg.

Post-capillary PH can further be divided into two subtypes: The first form, ipc-PH, can be explained by hemodynamic parameters and the fact that, in the context of a left ventricular overload, there is an increased congestion of blood into the left atrium and pulmonary veins. In the second form, cpc-PH, left ventricular loading triggers further congestion into the pulmonary arteries and consequent remodeling with constriction, inflammation, fibrosis, and endothelial activation [5]. Ipc-PH is determined by a DPG < 7 mmHg and/or a PVR ≤ 3 WU, and cpc-PH is associated with a DPG ≥ 7 mmHg and/or a PVR > 3 WU. The challenging aspect of this subdivision, according to currently valid guidelines, is that patients with an isolated elevation of a PVR > 3 WU or DPG ≥ 7 mmHg could be assigned to both ipc-PH and cpc-PH. These non-classifiable patient cases have already been the focus of intense scientific debates without a satisfactory problem resolution [6].

The purpose of this study was to evaluate the general prevalence of PH in patients undergoing TAVI, classify these patients based on RHC measurements in different PH subtypes, and analyze the prognostic and cut-off values of PH subtypes on survival after TAVI regarding one-year mortality.

## 2. Methods

### 2.1. Patient Population

The present study included 284 patients with symptomatic severe AS who underwent RHC examination at the University Hospital of Jena between 2010 and 2015 as part of a TAVI planning process. The indication for TAVI was made by a multidisciplinary heart-team consisting of cardiologists and cardiac surgeons. Patients that had not undergone RHC, with a diagnosis of moderate or severe mitral stenosis and under mechanical ventilation, were excluded. All examinations were carried out with the principles of the Declaration of Helsinki and Good Clinical Practice. The prospective study was approved by the local ethics committee of the University Hospital of Jena (Jena 3237-09/11).

### 2.2. Procedure of RHC

A few days before the TAVI procedure, various pressure measurements and hemodynamic parameters were obtained by an RHC. Pressure curves were measured and recorded using fluid-filled catheters connected to pressure transducers. Right atrial pressure, right ventricular pressure, systolic artery pressure (sPAP; mmHg), diastolic artery pressure (dPAP; mmHg), and mPAP (mmHg) were recorded. Additionally, determinations of mPCWP (mmHg), systolic vascular resistance (SVR; dyn × s × cm^−5^), PVR (dyn × s × cm^−5^), the transpulmonary gradient (TPG; mmHg), and DPG (mmHg) were carried out. DPD was calculated as the difference between dPAP and mPCWP, TPG as mPAP minus mPCWP, and PVR as TPG × 80/cardiac output (CO). CO was generated using the modified Fick method with estimated oxygen consumption and was indexed to the body surface area to calculate the cardiac index. For all calculations, Metek Software (Metek, Roetgen, Germany) was used.

### 2.3. Subtypes of PH

Patients were categorized in several PH subtypes due to RHC measurements according to ESC guidelines. An invasive mPAP < 25 mmHg led to the exclusion of PH and a mPAP ≥ 25 mmHg was conclusive with the diagnosis of PH. Patients with PH were further subdivided into prec-PH (mPCWP ≤ 15 mmHg) and post-capillary (mPCWP > 15 mmHg) subtypes. Lastly, post-capillary PHs were once again dichotomized into ipc-PH (DPG < 7 mmHg and/or PVR ≤ 3 WU) and cpc-PH (DPG ≥ 7 mmHg and/or PVR > 3 WU). In cases of values with a DPG < 7 mmHg and a PVR > 3 WU, the term “borderlinepc-PH” was introduced. Patients with a DPG ≥ 7 mmHg and a PVR ≤ 3 WU were not observed.

### 2.4. Transthoracic Echocardiography

Transthoracic echocardiography was performed using commercial ultrasonic devices (iE33 and Epic; Philips Healthcare). Before RHC performance, severe AS grading was confirmed according to the current guidelines of the ESC measuring the mean pressure gradient (MPG; mmHg) and peak pressure gradient (PPG; mmHg), maximum velocity (Vmax; m/s), and aortic valve area (AVA; cm^2^). Left ventricular ejection fraction (LVEF) was calculated via the Simpson method. Mitral, aortic, and tricuspid valve regurgitations were analyzed using spectral and color Doppler images and graded as minimal, mild (I), moderate (II), or severe (III). Maximal tricuspid regurgitant jet velocity, combined with central venous pressure using the diameter of inferior vena cava, was used to calculate sPAP.

### 2.5. TAVI Procedure

For transfemoral TAVI, a valve prosthesis of Edwards Sapien, CoreValve, or Jena Valve were implanted, whereas the transapical approach was performed with a prosthesis of Jena Valve or Edwards Sapien. For the closure of arterial and femoral access, 2 × Proglide (Abbott, Chicago, ILL, USA) was used. The pharmacological regimen after TAVI consisted of 100 mg acetylsalicylic acid and 75 mg clopidogrel for three months followed by lifetime medication with 100 mg acetylsalicylic acid as monotherapy.

### 2.6. Clinical Follow-Up and Study Endpoint

Clinical follow-up was performed at 7 days, 30 days, and 12 months after TAVI by clinical visit (7 days), telephone interview (30 days), and outpatient examination (12 months). Thirty-days and 1-year mortalities were the primary endpoints of this study. Secondary endpoints included major vascular complications, strokes, and myocardial infarction. Additionally, LVEF, sPAP, and mitral and tricuspid valve insufficiencies were analyzed by echocardiographic measurements. The New York Heart Association (NYHA) score was used for clinical assessment.

### 2.7. Statistical Analysis

Nominal and ordinal variables are presented as frequencies/percentages and metric variables as mean ± standard deviation. Baseline characteristics of different PH subtypes were compared using an analysis of the chi-square test for categorical data or analysis of variance (ANOVA) for metric data. Event-free survival was carried out with the Kaplan–Meier method and the curves were compared using the log-rank test. The univariate Cox proportional hazard regression model was used to calculate the hazard ratio (HR) with a 95% confidence interval (CI) for several influencing factors associated with 1-year mortality in patients undergoing the TAVI procedure. Afterwards, multivariable Cox regression was performed to assess independent predictors of mortality. Therefore, covariates associated with mortality in the univariate analysis (*p <* 0.100) were entered, and a backward variable elimination was carried out. To determine an optimal cut-off value according to the overall survival and RHC measurements in different PH subtypes, receiver operator characteristic (ROC) curves with an area under the curve (AUC) analysis and a separate analysis of the Youden index (YI) were performed. A *p*-value of ≤0.050 was considered statistically significant. Statistical analyses were performed using SPSS 25 (SPSS Inc, Chicago, IL, USA).

## 3. Results

### 3.1. PH Subtypes of Study Collective

**Figure 1:**
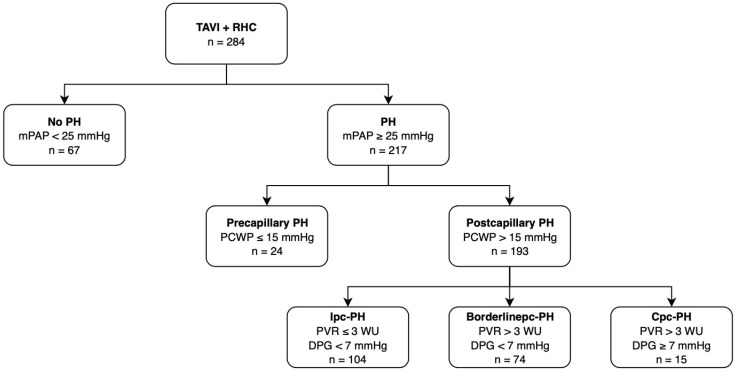
Patient disposition in study cohort. TAVI: transcatheter aortic valve implantation; RHC: right heart catheterization; PH: pulmonary hypertension; mPAP: mean pulmonary arterial pressure; prec-PH: pre-capillary pulmonary hypertension; ipc-PH: isolated post-capillary pulmonary hypertension; borderlinepc-PH: borderline post-capillary pulmonary hypertension; cpc-PH: combined post- and pre-capillary pulmonary hypertension.

The subdivision of PH according to the hemodynamic measurements of the currently valid 2015 ESC guidelines is summarized in Figure 1.

A total of 284 patients with high-grade AS planning for TAVI and an existing RHC were included in the study. Sixty-seven patients (23.6%) did not suffer from concomitant PH, whereas 217 (76.4%) fulfilled the criteria of PH. Of these, 24 were recorded with prec-PH, 104 with ipc-PH, 74 with so-called “borderlinepc-PH”, and 15 with cpc-PH.

### 3.2. General Characteristics

The general data, percentual distributions of concomitant diseases, echocardiographic measurements, measurements of RHC, and procedural TAVI data are shown in Table 1.

In the overall cohort, patients showed a mean age of 80.5 ± 7.2 years and a EuroScore II of 6.7 ± 5.4. Regarding the medical history, 34.5% had non-insulin-dependent diabetes mellitus, 91.2% arterial hypertension, 25% COPD, and 14.8% myocardial infarction. Additionally, an average EF of 56.6 ± 16.2%, AVA of 0.65 ± 0.19 cm^2^, and maximum velocity (AV Vmax) of 4.3 ± 0.7 m/s were documented.

A further subdivision of PH revealed significant differences between subgroups in terms of weight, percentage distribution of non-insulin-dependent diabetes mellitus, atrial fibrillation, LVEF, sPAP, AVA, and all RHC measurements.

### 3.3. Kaplan–Meier Curves and Cox Proportional Hazard Regression

**Figure 2:**
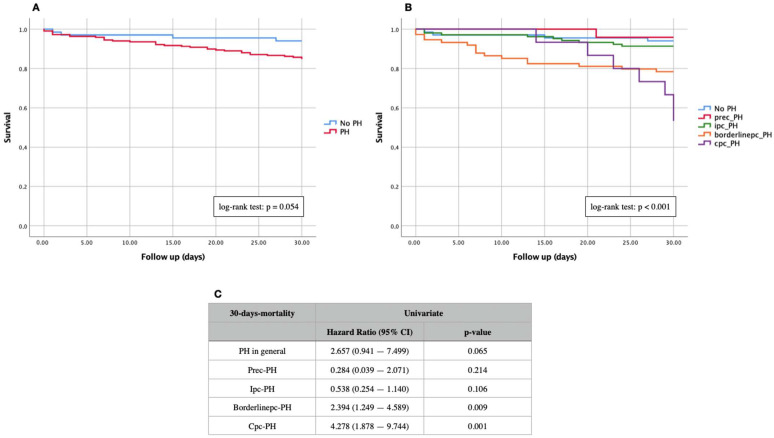
Kaplan–Meier curves of 30-days mortality in patients with severe AS. (**A**) Comparison of non-PH vs. PH; (**B**) comparison of non-PH and different PH subtypes; (**C**) univariate Cox regression analysis of different PH subtypes. Prec-PH: pre-capillary pulmonary hypertension; Ipc-PH: isolated post-capillary pulmonary hypertension; borderlinepc-PH: borderline post-capillary pulmonary hypertension; Cpc-PH: combined post- and pre-capillary pulmonary hypertension.

**Figure 3:**
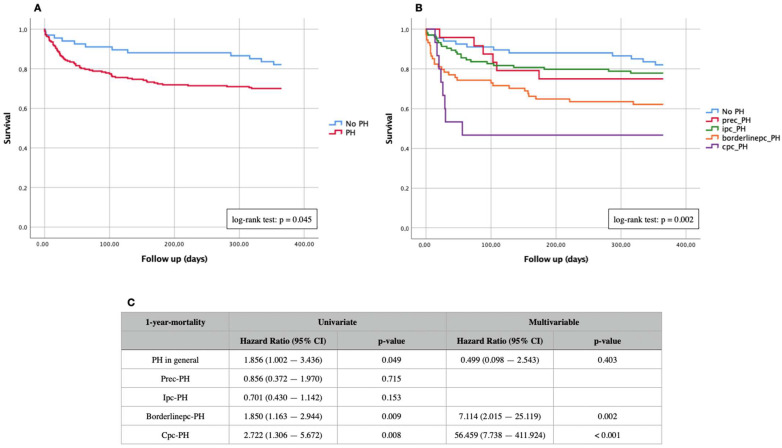
Kaplan–Meier curves of 1-year mortality in patients with severe AS. (**A**) Comparison of non-PH vs. PH; (**B**) comparison of non-PH and different PH subtypes; (**C**) univariate and multivariable Cox regression analyses of different PH subtypes. Prec-PH: pre-capillary pulmonary hypertension; Ipc-PH: isolated post-capillary pulmonary hypertension; Borderlinepc-PH: borderline post-capillary pulmonary hypertension; Cpc-PH: combined post- and pre-capillary pulmonary hypertension.

**Figure 4:**
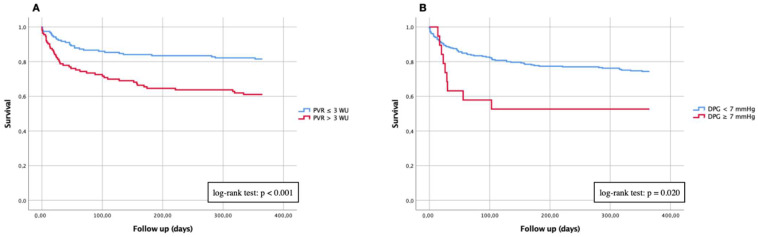
Kaplan–Meier curves of 1-year mortality in patients with severe AS and different expression levels of PVR and DPG. (**A**) Comparison of patients with PVR ≤ 3 WU vs. PVR > 3 WU; (**B**) comparison of patients with DPG < 7 mmHg vs. DPG ≥ 7 mmHg. PVR: pulmonary vascular resistance; DPG: diastolic pressure gradient.

Kaplan–Meier curves of “PH“ vs. “No PH“ and of different PH subtypes for primary endpoints (30-days and 1-year mortality) are demonstrated in Figure 2 (30-days mortality) and Figure 3 (1-year mortality). In addition, Kaplan–Meier analyses of the entire cohort were independently performed on the PH subtypes with different expression levels of PVR and DPG (Figure 4). Summaries of univariate and multivariable Cox proportional hazard regression analyses are presented in Table 2.

During an observation period of 30 days, 37 patients died in the entire study cohort; this corresponds to a percentage of 13.0. Of these, 33 patients (15.2%) had an invasively measured PH, and four patients (6.0%) had no PH. Neither a log-rank test (*p* = 0.054) (Figure 2A) nor a Cox hazard regression analysis (HR 2.657; 95% CI 0.941–7.499; *p* = 0.065) showed a statistical significance between the groups. Of the 33 deceased patients with PH, a further division by subtype went as follows: one patient with prec-PH (4.2%), nine with ipc-PH (8.7%), sixteen with borderlinepc-PH (21.6%), and seven with cpc-PH (46.7%). In contrast, the Kaplan–Meier curve with respect to 30-days mortality divided into subtypes (Figure 2B) showed significant differences in the log-rank test with *p* = 0.001. Borderlinepc-PH patients (HR 2.394; 95% CI 1.249–4.589; *p* = 0.009) and cpc-PH patients (HR 4.278; 95% CI 1.878–9.744; *p* = 0.001) demonstrated a significantly worse survival prognosis in the associated Cox hazard regression analysis (Figure 2C).

During a follow-up period of 12 months, the primary endpoint death was reached a total of 77 times (27.1%)—12 patients without hemodynamic evidence of PH (17.9%) and 65 patients with PH (30%). A log-rank test with *p* = 0.045 revealed a significant difference in adverse events between PH patients vs. non-PH patients (Figure 3A) as well as the result of the Cox hazard regression analysis (HR 1.856; 95% CI 1.002–3.436; *p* = 0.049). Of the 65 PH patients who died, six had prec-PH (25.0%), twenty-three ipc-PH (22.1%), twenty-eight borderlinepc-PH (37.8%), and eight cpc-PH (53.3%). Again, the Kaplan–Meier curve with division into various PH subtypes (Figure 3B) showed a significant difference in the log-rank test with *p* = 0.002.

To better assess the PVR and DPG data obtained by right heart catheterization with respect to 1-year survival, separate Kaplan–Meier curves were performed for the known cut-off values relevant for classification into the different PH groups. At 12 months after successful TAVI, 29/164 patients from the entire group died with a PVR ≤ 3 WU (Figure 4A). In contrast, there were 44/120 patients with a PVR > 3 WU who died significantly more often (log-rank test: *p* < 0.001). A similar analysis using the DPG showed that 68/265 patients with a DPG < 7 mmHg and 9/19 patients with a DPG ≥ 7 mmHg were no longer alive after 12 months (Figure 4B). Again, this showed a statistically significant difference with a log-rank test of *p* = 0.020.

The univariate Cox hazard regression analysis demonstrated a significantly increased 1-year mortality in patients suffering from borderlinepc-PH (HR 1.850; 95% CI 1.163–2.944; *p* = 0.009) or cpc-PH (HR 2.722; 95% CI 1.306–5.672; *p* = 0.008) compared with patients with prec-PH or ipc-PH (Figure 3C).

Further influencing factors associated with an increased 1-year mortality after TAVI were C-reactive protein concentrations (HR 1.006; 95% CI 1.000–1.011; *p* = 0.039), an STS-Score (HR 1.164; 95% CI 1.093–1.240; *p* < 0.001), EuroScore II (HR 1.081; 95% CI 1.041–1.123; *p* < 0.001), sPAP (HR 1.028; 95% CI 1.012–1.044; *p* < 0.001), AVA (HR 0.231; 95% CI 0.055–0.967; *p* = 0.045), and with the exception of DPG and CO—all collected RHC measurements. COPD (HR 1.939; 95% CI 1.218–3.085; *p* = 0.005), myocardial infarction (HR 1.782; 95% CI 1.040–3.054; *p* = 0.036), and stroke (HR 1.959; 95% CI 1.166–3.291; *p* = 0.011) in premedical history also represented significant influencing factors regarding shorter survival times.

All univariate covariates with *p* < 0.100 were used to perform a multivariable Cox regression analysis with a backward elimination. Lastly, borderlinepc-PH (HR 7.114; 95% CI 2.015–25.119; *p* = 0.002) and ipc-PH (HR 56.459; 95% CI 7.738–411.924; *p* < 0.001) were described as independent predictors of 1-year mortality, as well as a tricuspid regurgitation > II, EuroScore II, dPAP, and vascular complications. PVR and DPG, despite the significant mortality curves, were ultimately not isolated factors in the Cox regression analysis, resulting in increased mortality rates in patients with severe AS and TAVI.

### 3.4. ROC Curves and Cut-Off Values

In order to establish the sensitivity and specificity of RHC measurements for the detection of PH in the dependency of 1-year mortality, ROC curves, AUC, and cut-off values were determined for the overall cohort and for the respective PH subtypes. The results are summarized in Table 3.

ROC curves with the associated AUC of the overall cohort showed almost continuously significant *p*-values, with the exception of TPG, DPG, and CO. Especially a sPAP ≥ 49.50 mmHg (AUC 0.620; *p* = 0.002; YI 0.22), mPAP ≥ 34.50 mmHg (AUC 0.623; *p* = 0.001; YI 0,22), dPAP ≥ 15.50 mmHg (AUC 0.599; *p* = 0.010; YI 0.19), mPCWP ≥ 16.50 mmHg (AUC 0.586; *p* = 0.036; YI 0.16), and PVR ≥ 3.15 WU (AUC 0.631; *p* = 0.001; YI 0.26) were associated with an earlier death.

After classification into PH subtypes, significant differences could only be observed in patients with cpc-PH with respect to sPAP (cut-off value 67.00 mmHg; AUC 0.857; *p* = 0.021; YI 0.86), mPAP (cut-off value 43.50 mmHg; AUC 0.821; *p* = 0.037; YI 0.71), DPG (cut-off value 9.50 mmHg; AUC 0.821; *p* = 0.037; YI 0.61), and TPG (cut-off value 23.50 mmHg; AUC 0.898; *p* = 0.013; YI 0.86).

## 4. Discussion

### 4.1. PH as a Solitary Risk Factor in Patients Receiving TAVI?

The prevalence of PH in patients with severe AS ranges from 11–56% according to several studies [7,8]. In the present study, 217 of 284 patients were diagnosed with an mPAP of ≥25 mmHg by RHC, thus fulfilling the essential diagnostic criterion of PH. This corresponded to a frequency of 76.4% and a similar percentage distribution as in O’Sullivan et al. [9], who reported nearly 75% in a study on TAVI patients with RHC measurements.

Several publications have shown that PH affects the outcome and survival in patients with severe AS. In particular, Luçon et al. [10] demonstrated during the evaluation of the FRANCE-2 registry that patients who were PH–defined echocardiographically by an sPAP ≥ 40 mmHg had an increased 1-year mortality in comparison with non-PH patients. The invasively obtained PH data of our study were not only in this respect in agreement with Luçon et al., but also with regard to the fact that no significant difference in 30-days survival was observed. However, in the univariate Cox hazard regression analysis, the presence of PH was shown to be an independent predictor of earlier death in the period of one year.

Not only the presence or absence of PH but also the appropriate subgrouping were essential for patient survival after TAVI. This thesis was confirmed by O’Sullivan et al., who proved that patients with subtypes of prec-PH and cpc-PH, but not ipc-PH, showed a higher 1-year mortality, whereas Schewel et al. [11] found higher mortality rates after 1 year in prec-PH and ipc-PH patients but not in cpc-PH patients. Another equally different result was reached by Weber et al. [12] who, in contrast to Schewel and colleagues, not only demonstrated significant differences within subgroups, but also a significantly higher mortality in patients with cpc-PH in comparison to those in other PH subtypes. Our prospective study shared Weber’s assessment of both an increased mortality in the cpc-PH and cpc-PH subtypes as an independent predictor of death in the multivariable Cox hazard regression analysis.

O’Sullivan, Weber, Schewel and this current study all have in common that the classification of TAVI patients into PH subgroups was done according to invasive RHC measurements. Nevertheless, inconsistent results regarding 1-year mortality occurred. This may have been because different definitions and criteria of PH were consulted, and no uniform classification was carried out. For example, O’Sullivan focused on PVR alone to differentiate between ipc- and cpc-PH, and in our study, we tried to break new ground by establishing the borderline subtype.

### 4.2. Prec-PH and AS—Does This Constellation Fit Together?

By strictly applying ESC guidelines regarding hemodynamic measurements (mPAP ≥ 25 mmHg, mPCWP ≤ 15 mmHg), 11.1% of all PH patients (24/217) with severe AS in the present study were diagnosed with prec-PH. This percentage distribution seamlessly integrated in a row with similar proportions of further TAVI studies, for example, Kaple et al. [13] who described a prec-PH cohort of nearly 12%, O’Sullivan et al. who reported 13%, and Weber et al. who documented 12.9%. Nearly 30% of our patients suffered from COPD; other previous medical diagnoses such as idiopathic PH, pulmonary fibrosis, chronic pulmonary embolism, or rheumatic diseases could be excluded as a cause of prec-PH.

In contrast to O’Sullivan and Schewel et al., who found significantly worse clinical outcomes and less functional improvements regarding 1-year mortality in TAVI patients with prec-PH, Weber et al. and our study showed no significant differences compared with non-PH and ipc-PH patients, respectively. This could be because LVEDP instead of mPCWP was used as a classification basis in the two studies. In a clinical setting, both measures are used more-or-less interchangeably to obtain information about left ventricular filling pressure. However, previous studies have indicated that due to several variables, such as mitral regurgitation, atrial fibrillation, and diastolic dysfunction [14], an unrestricted equality of both measurements was not purposeful and led to misclassifications [15,16]. Hemnes et al. [17] additionally observed in elderly patients in their cohort a general underestimation of mPCWP, especially in the limited values of 15 mmHg, in which case an erroneously precapillary rather than postcapillary allocation occurred. This could also explain the results of present study, that by a close inspection of the Kaplan–Meier curves, similar curve progressions were present with respect to 30- and 365-days mortality. Moreover, many patients suffering from severe AS are pretreated with diuretic drugs; therefore, a strictly adjusted fluid balance may be the reason for post-capillary PH, even if the LVEDP or mPCWP are measured at <15 mmHg.

Furthermore, in PH due to left heart disease, the DPG has been proposed as a specific marker of pre-capillary involvement [18]. In this regard, Gerges et al. postulated that an optimized classification of prec-PH would be to introduce a DPG ≥ 7 mmHg as an additional diagnostic criterion in addition to mPAP and mPCWP. In our study, this would have led to the exclusion of 22 patients suffering from prec-PH. The mean DPG was 3.6 ± 2.7 mmHg, which nearly as low as Weber’s study group (0–5 mmHg). In comparison, the cohort of O’Sullivan (8.4 ± 6.9 mmHg), and also that of Schewel 19.5 ± 5.9 mmHg, showed a significantly higher mean DPG. Considering the different prec-PH survival curves of O’Sullivan and Schewel compared with those of Weber and our study, DPG should be given a relevant place in the classification of prec-PH to prevent misclassification and thus overtreatment.

### 4.3. Borderlinepc-PH—Is It Worth Being a New Subtype?

Patients with a DPG ≤ 7 mmHg and PVR > 3 WU were deliberately treated as a separate subgrouping in this study to investigate patients with postcapillary PH as a separate entity who could not be adequately classified into either the ipc-PH or cpc-PH subtypes according to the currently valid ESC guidelines. For this purpose, the term “borderlinepc-PH” was defined. Patients with a constellation DPG > 7 mmHg and PVR ≤ 3 WU were not observed.

Hemodynamically, this group can best be classified as an intermediate state between ipc-PH and cpc-PH [19], whereby left ventricular blood congestion have already reached pulmonary arteries, but no irreversible remodeling processes have yet been initiated. This intermediate stage could likewise be transferred with regard to Kaplan–Meier curves in the present study, as the borderlinepc-PH curve was reflected between that of the ipc-PH and cpc-PH subtypes. Moreover, in the RHC measurements, a continuous increase was shown in the sequence ipc → borderline → cpc with respect to the parameters: sPAP, mPAP, dPAP, DPG, PVR, and TPG. Thus, the borderline type took a middle position. An association was observed in Caravita et al. [20], who published similar survival curves and comparably invasive hemodynamic profiles of the different types (borderline corresponded here to the intermediate group). In the present study, borderlinepc-PH patients showed a higher mPCWP with a concomitant low CO compared with ipc-PH or cpc-PH patients. With a simultaneous lower ejection fraction of 52% on average compared with the other subtypes, the suspected diagnosis of the concomitant HFpEF constellation was confirmed. 

Clinically relevant was the fact that in the multivariable Cox hazard regression analysis, not only the cpc-PH but also the borderlinepc-PH types were detected as independent predictors for an increased 1-year mortality. This confirmed the hypothesis of Palazzini et al. that in post-capillary PH patients, an isolated increase in a PVR > 3 WU was associated with a worse long-term prognosis. The significant cut-off value of 3 to 15 WU in the overall cohort provided further confirmation of the guideline threshold. Nevertheless, a 1-year mortality in TAVI patients increased if, in addition to a PVR > 3 WU, a DPG ≥ 9.50 mmHg was present. This corresponded to a cpc-PH constellation. The DPG itself is highly controversial as a parameter [21] and should be deleted without replacement as a classification criterion for post-capillary PH according to the 6th World Symposium on PH of 2018 [22]. Nevertheless, as described by Gerges et al., in both “isolated” prec-PH and cpc-PH, the DPG is an important marker of pulmonary vascular remodeling processes.

In the end, it is a legitimate question to ask whether a further subdivision of post-capillary PH into three rather than two subtypes is useful. In view of previous studies and our own study results, we answer “yes” to this question. We assume that irreversible pulmonary remodeling processes are primarily observed in cpc-PH patients and that the borderlinepc-PH stage is therefore an important transitional stage to prevent these structural changes, e.g., with prompt TAVI. Therefore, an RHC is and remains an important diagnostic tool, which should be used in particular when clinical symptoms, echocardiographic data, and spiroergometric results indicate relevant PH in severe AS in order to evaluate risk constellations.

## 5. Conclusions

By establishing borderlinepc-PH defined with a mPAP ≥ 25 mmHg, mPCWP > 15 mmHg, DPG < 7 mmHg, and PVR > 3 WU, we tried to compensate for the discrepancy in the current ESC guideline. We showed that in patients with severe AS, an intermediate stage was generated by establishing a third postcapillary subtype, which was an intermediate between ipc-PH and cpc-PH in terms of survival curves and RHC diagnostic results. The extent to which irreversible remodeling processes of the pulmonary vascular structures can be stopped by prompt treatment, e.g., by TAVI, should be investigated in larger study populations with invasive RHC data before and after TAVI.

## 6. Limitations

This study relied on data from a single center and may not have reflected general practice. Furthermore, we must consider technical pitfalls occurring in everyday hemodynamic testing, although the assessments were performed by experienced physicians proficient in cardiac hemodynamics. Additionally, the fluid statuses of patients, e.g., volume overload or dehydration due to excessive diuretic therapy, can falsify invasive hemodynamic parameters.

## Figures and Tables

**Table 1:** General characteristics, echocardiographic measurements, and invasive hemodynamic profiles of study cohort.

	Overall Cohort		No PH		Prec-PH		Ipc-PH		Borderlinepc-PH		Cpc-PH		
	Mean	SD (±)	Mean	SD (±)	Mean	SD (±)	Mean	SD (±)	Mean	SD (±)	Mean	SD (±)	*p*-Value
**General data**													
Age (years)	80.5	7.2	80.6	6.2	83.5	5.6	79.6	8.1	80.6	7.1	80.6	7.4	0.234
Weight (kg)	73.7	13.1	70.8	13.1	75.4	16.3	77.2	12.6	71.5	12.1	70.1	10.9	0.005
Height (cm)	164.4	8.9	162.9	8.9	164.7	9.0	166.0	8.8	163.1	8.5	166.0	9.6	0.102
Creatinine (µmol/L)	121.9	87.8	104.6	54.4	114.6	53.2	136.4	109.7	121.8	90.6	111.1	52.2	0.255
C-reactive protein (mg/L)	18.9	32.8	10.8	22.1	15.5	19.8	19.6	34.3	26.2	41.5	19.5	27.4	0.132
Hemoglobin (mmol/L)	7.5	1.1	7.5	1.0	7.7	0.9	7.2	1.1	7.5	1.2	7.7	1.4	0.218
STS-Score	4.1	2.5	3.5	2.1	4.7	3.0	4.1	2.8	4.3	2.2	4.0	2.8	0.267
EuroScore II	6.7	5.4	5.0	5.7	6.0	4.2	7.4	5.2	7.6	5.3	7.4	5.1	0.175
**Concomitant diseases**													
Insulin-dependent diabetes (%)	23.9		19.4		8.3		29.8		25.7		20.0		0.186
Non-insulin-dependent diabetes (%)	34.5		35.8		50.0		25.0		36.5		60.0		0.024
Arterial hypertension (%)	91.2		92.5		95.8		88.5		91.9		93.3		0.756
Coronary heart disease - 1 vessel (%)	21.8		20.9		16.7		23.1		21.6		26.7		0.950
Coronary heart disease - 2 vessels (%)	13.4		10.4		25.0		15.4		12.2		6.7		0.194
Coronary heart disease - 3 vessels (%)	15.5		14.9		4.2		13.5		23.0		13.3		0.206
COPD (%)	25.0		19.4		29.2		27.9		23.0		33.3		0.641
Myocardial infarction (%)	14.8		14.9		4.2		17.5		14.9		13.3		0.599
Stroke (%)	15.1		10.4		20.8		19.2		12.2		13.3		0.454
Atrial fibrillation (%)	52.6		34.9		40.0		66.7		52.4		71.4		0.023
NYHA II	11.3		17.9		20.8		8.7		5.4		13.3		0.072
NYHA III	60.2		62.7		62.5		58.7		62.2		46.7		0.844
NYHA IV	22.9		13.4		12.5		25.0		29.7		33.3		0.093
**Echocardiographic measurements**													
EF (%)	56.6	16.2	60.2	15.1	59.8	13.2	56.8	16.0	52.0	17.4	57.2	16.7	0.041
LVEDD (mm)	48.9	8.0	47.2	7.9	48.1	7.3	49.9	7.5	49.5	8.4	49.3	9.9	0.284
LVESD (mm)	32.5	9.5	30.0	9.3	32.6	9.6	33.0	9.3	33.9	9.5	34.8	11.6	0.286
sPAP (mmHg)	41.8	13.4	32.6	7.7	34.3	11.6	44.0	12.1	47.0	14.7	50.3	13.5	<0.001
AVA (cm^2^)	0.7	0.2	0.7	0.2	0.6	0.2	0.7	0.2	0.6	0.2	0.7	0.2	0.012
AV max (m/s)	4.3	0.7	4.5	0.7	4.4	0.5	4.3	0.6	4.2	0.8	4.4	0.8	0.243
AV dpmax (mmHg)	78.5	26.3	82.8	25.6	79.4	15.5	75.1	23.0	78.2	32.3	81.1	30.2	0.470
AV dpmean (mmHg)	47.7	16.7	50.6	17.5	48.2	12.5	45.6	14.2	47.6	19.9	48.0	17.9	0.481
Mitral regurgitation I° (%)	49.6		63.6		62.5		45.5		32.4		66.7		<0.001
Mitral regurgitation II° (%)	38.4		31.8		16.7		41.4		48.6		20.0		0.042
Mitral regurgitation III° (%)	8.4		3.1		16.7		9.1		14.9		6.7		0.076
Tricuspid regurgitation I° (%)	51.9		61.3		45.8		48.4		48.6		33.3		0.284
Tricuspid regurgitation II° (%)	32.7		30.6		29.2		32.3		29.7		46.7		0.791
Tricuspid regurgitation III° (%)	12.3		3.2		20.8		17.1		16.2		13.3		0.028
Paravalvular regurgitation I° (%)	37.9		38.1		60.0		39.6		30.0		37.5		0.524
Paravalvular regurgitation II° (%)	13.1		11.9		10.0		15.1		10.0		25.0		0.798
Paravalvular regurgitation III° (%)	0.7		0.0		10.0		0.0		0.0		0.0		0.006
**Measurements of RHC**													
sPAP (mmHg)	53.5	18.1	33.8	6.8	44.3	7.5	55.9	14.1	67.1	14.2	72.4	19.8	<0.001
mPAP (mmHg)	34.1	12.0	19.6	3.7	27.6	2.9	36.3	8.2	42.9	8.7	50.1	11.3	<0.001
dPAP (mmHg)	20.1	8.8	9.9	4.1	16.5	2.6	22.2	6.8	24.7	6.7	33.5	7.8	<0.001
mPCWP (mmHg)	21.3	9.4	10.6	4.0	12.9	1.8	25.5	7.5	27.1	6.9	23.7	7.5	<0.001
DPG (mmHg)	−1.2	5.7	−0.7	4.8	3.6	2.7	−3.4	4.4	−2.4	5.5	10.1	3.3	<0.001
PVR (WU)	3.3	2.1	2.5	1.5	3.2	1.0	1.9	0.6	5.0	2.2	6.7	2.3	<0.001
TPG (mmHg)	12.8	6.1	8.9	3.3	14.8	2.9	10.8	3.6	15.8	5.6	27.0	9.1	<0.001
CO (L/min)	4.2	1.2	4.2	1.3	4.2	1.0	4.5	1.2	3.7	1.0	3.9	1.0	<0.001
**Procedurale data**													
Transfemoral approach (%)	75.4		77.6		83.3		72.1		77.0		66.7		0.678
CoreValve (%)	22.3		17.9		29.2		25.0		21.6		20.0		0.800
JenaValve (%)	15.9		16.4		12.5		15.4		16.2		20.0		0.979
Edwards (%)	61.8		65.7		58.3		59.6		62.2		60.0		0.941
Major vascular complications (%)	11.3		11.9		8.3		9.6		12.2		20.0		0.786

EF: ejection fraction; LVEDD: left ventricular end diastolic diameter; LVESD: left ventricular end systolic diameter; sPAP: systolic pulmonary arterial pressure; AVA: aortic valve area; AV max: maximal velocity over aortic valve; AV dpmax: maximal pressure gradient over aortic valve; AV dpmean: mean pressure gradient over aortic valve; mPAP: mean pulmonary arterial pressure; dPAP: diastolic pulmonary arterial pressure; mPCWP: mean pulmonary capillary wedge pressure; DPG: diastolic pressure gradient; TPG: transpulmonary pressure gradient; CO: cardiac output.

**Table 2:** Univariate and multivariable predictors of 1-year survival in pulmonary hypertension due to severe AS.

	Univariate		Multivariable	
	Hazard Ratio (95% CI)	*p*-Value	Hazard Ratio (95% CI)	*p*-Value
**General data**				
Age (years)	0.998 (0.967–1.030)	0.894		
Weight (kg)	0.989 (0.972–1.007)	0.216		
Height (cm)	1.000 (0.974–1.025)	0.974		
Creatinine (µmol/L)	1.001 (0.999–1.003)	0.247		
C-reactive protein (mg/L)	1.006 (1.000–1.011)	0.039	1.000 (0.984–1.016)	0.968
Hemoglobin (mmol/L)	0.899 (0.720–1.124)	0.351		
STS-Score	1.164 (1.093–1.240)	<0.001	1.114 (0.951–1.306)	0.181
EuroScore II	1.081 (1.041–1.123)	<0.001	1.138 (1.049–1.235)	0.002
**Concomitant diseases**				
Insulin-dependent diabetes	1.392 (0.854–2.268)	0.184		
Non-insulin-dependent diabetes	1.366 (0.866–2.155)	0.179		
Arterial hypertension	1.417 (0.572–3.508)	0.451		
Coronary heart disease ≥ 2 vessels	1.354 (0.844–2.171)	0.209		
COPD	1.939 (1.218–3.085)	0.005	1.207 (0.380–3.836)	0.750
Myocardial infarction	1.782 (1.040–3.054)	0.036	1.202 (0.305–4.730)	0.792
Stroke	1.959 (1.166–3.291)	0.011	0.367 (0.079–1.718)	0.203
Atrial fibrillation	1.619 (0.869–3.019)	0.129		
NYHA ≥ III	1.951 (0.786–4.843)	0.150		
**Echocardiographic measurements**				
EF (%)	0.990 (0.976–1.003)	0.135		
LVEDD (mm)	1.015 (0.988–1.044)	0.273		
LVESD (mm)	1.004 (0.974–1.035	0.788		
sPAP (mmHg)	1.028 (1.012–1.044)	<0.001	0.974 (0.928–1.022)	0.285
AVA (cm^2^)	0.231 (0.055–0.967)	0.045	0.268 (0.014–5.192)	0.384
AV max (m/s)	0.846 (0.605–1.182)	0.327		
AV dpmax (mmHg)	0.995 (0.985–1.004)	0.249		
AV dpmean (mmHg)	0.992 (0.978–1.006)	0.267		
Mitral regurgitation ≥ II°	1.319 (0.841–2.069)	0.229		
Tricuspid regurgitation ≥ II°	1.890 (1.166–3.065)	0.010	3.119 (1.217–7.994)	0.018
Paravalvular regurgitation ≥ II°	1.720 (0.792–3.733)	0.170		
**Measurements of RHC**				
sPAP (mmHg)	1.019 (1.008–1.030)	0.001	1.015 (0.974–1.058)	0.480
mPAP (mmHg)	1.032 (1.014–1.051)	0.001	1.026 (0.874–1.203)	0.756
dPAP (mmHg)	1.034 (1.009–1.059)	0.008	0.912 (0.841–0.990)	0.027
mPCWP (mmHg)	1.024 (1.001–1.047)	0.041	1.043 (0.931–1.170)	0.467
DPG (mmHg)	1.015 (0.973–1.059)	0.480		
PVR (WU)	1.167 (1.077–1.265)	<0.001	1.079 (0.867–1.344)	0.494
PVR > 3 (WU)	2.414 (1.510–3.859)	<0.001	0.492 (0.093–2.615)	0.406
CO (L/min)	0.859 (0.697–1.060)	0.156		
**Procedurale data**				
Transfemoral approach	0.540 (0.338–0.862)	0.010	0.546 (0.230–1.297)	0.171
CoreValve	0.990 (0.578–1.696)	0.971		
JenaValve	1.681 (0.981–2.880)	0.059	0.478 (0.121–1.882)	0.291
Edwards	0.702 (0.448–1.099)	0.121		
Major vascular complications	3.351 (1.953–5.749)	<0.001	4.194 (1.272–13.829)	0.019

EF: ejection fraction; LVEDD: left ventricular end diastolic diameter; LVESD: left ventricular end systolic diameter; sPAP: systolic pulmonary arterial pressure; AVA: aortic valve area; AV max: maximal velocity over aortic valve; AV dpmax: maximal pressure gradient over aortic valve; AV dpmean: mean pressure gradient over aortic valve; mPAP: mean pulmonary arterial pressure; dPAP: diastolic pulmonary arterial pressure; mPCWP: mean pulmonary capillary wedge pressure; DPG: diastolic pressure gradient; TPG: transpulmonary pressure gradient; CO: cardiac output.

**Table 3:** Cut-off values, ROC curves, AUC analysis, and Youden indices of RHC measurements concerning overall cohort and different PH subtypes.

	AUC	*p*-Value	Cut-Off	Sensitivity	Specificity	Youden Index
**Measurements of RHC-overall cohort**						
sPAP (mmHg)	0.620	0.002	49.50	0.69	0.53	0.22
mPAP (mmHg)	0.623	0.001	34.50	0.62	0.60	0.22
dPAP (mmHg)	0.599	0.010	15.50	0.79	0.60	0.19
mPCWP (mmHg)	0.586	0.036	16.50	0.77	0.40	0.16
DPG (mmHg)	0.511	0.772	1.50	0.36	0.73	0.09
PVR (WU)	0.631	0.001	3.15	0.59	0.67	0.26
TPG (mmHg)	0.575	0.053	12.50	0.55	0.60	0.16
CO (L/min)	0.431	0.082	2.45	0.99	0.05	0.03
**Measurements of RHC-prec-PH**						
sPAP (mmHg)	0.519	0.894	37.50	1.00	0.17	0.17
mPAP (mmHg)	0.602	0.463	30.50	0.33	0.94	0.28
dPAP (mmHg)	0.611	0.424	15.50	0.83	0.50	0.33
mPCWP (mmHg)	0.486	0.920	14.50	0.33	0.83	0.17
DPG (mmHg)	0.542	0.764	8.50	0.17	1.00	0.17
PVR (WU)	0.711	0.157	3.30	1.00	0.67	0.67
TPG (mmHg)	0.551	0.714	13.50	0.83	0.33	0.17
CO (L/min)	0.267	0.118	2.95	1.00	0.06	0.06
**Measurements of RHC-ipc-PH**						
sPAP (mmHg)	0.562	0.366	47.50	0.87	0.32	0.19
mPAP (mmHg)	0.551	0.457	33.50	0.74	0.48	0.22
dPAP (mmHg)	0.553	0.440	27.50	0.30	0.84	0.14
mPCWP (mmHg)	0.530	0.661	19.50	0.87	0.36	0.13
DPG (mmHg)	0.523	0.739	-5.50	0.87	0.32	0.19
PVR (WU)	0.449	0.475	2.08	0.48	0.56	0.03
TPG (mmHg)	0.511	0.876	7.50	0.91	0.19	0.10
CO (L/min)	0.395	0.141	7.40	0.10	1.00	0.09
**Measurements of RHC-borderlinepc-PH**						
sPAP (mmHg)	0.514	0.854	81.50	0.29	0.91	0.20
mPAP (mmHg)	0.513	0.854	50.50	0.29	0.89	0.18
dPAP (mmHg)	0.467	0.636	12.00	1.00	0.06	0.06
mPCWP (mmHg)	0.490	0.889	37.50	0.14	0.96	0.10
DPG (mmHg)	0.511	0.872	1.50	0.32	0.78	0.10
PVR (WU)	0.574	0.290	4.22	0.68	0.52	0.20
TPG (mmHg)	0.535	0.614	19.50	0.32	0.82	0.14
CO (L/min)	0.557	0.413	3.19	0.75	0.41	0.16
**Measurements of RHC-cpc-PH**						
sPAP (mmHg)	0.857	0.021	67.00	1.00	0.86	0.86
mPAP (mmHg)	0.821	0.037	43.50	1.00	0.71	0.71
dPAP (mmHg)	0.643	0.355	38.50	0.50	0.86	0.36
mPCWP (mmHg)	0.482	0.908	16.50	1.00	0.14	0.14
DPG (mmHg)	0.821	0.037	9.50	0.75	0.86	0.61
PVR (WU)	0.729	0.156	4.74	1.00	0.50	0.50
TPG (mmHg)	0.898	0.013	23.50	1.00	0.86	0.86
CO (L/min)	0.429	0.643	2.92	0.88	0.29	0.16

sPAP: systolic pulmonary arterial pressure; mPAP: mean pulmonary arterial pressure; dPAP: diastolic pulmonary arterial pressure; mPCWP: mean pulmonary capillary wedge pressure; DPG: diastolic pressure gradient; TPG: transpulmonary pressure gradient; CO: cardiac output.

## Data Availability

The data presented in this study are available on request from the corresponding author.
